# Evidence supporting the use of a brief cognitive assessment in routine clinical assessment for psychosis

**DOI:** 10.1038/s41537-022-00322-z

**Published:** 2022-12-17

**Authors:** M. Cowman, E. Lonergan, T. Burke, C. R. Bowie, A. Corvin, D. W. Morris, K. O’Connor, G. Donohoe

**Affiliations:** 1 Centre for Neuroimaging, Cognition & Genomics (NICOG), School of Psychology, National University of Ireland Galway, Galway, Ireland; 2 First Episode Psychosis Service, South Lee Mental Health Service, Cork, Ireland; 3 Department of Psychology, Queen’s University, Kingston, ON Canada; 4 Department of Psychiatry, Trinity College Dublin, Dublin, Ireland

**Keywords:** Psychosis, Working memory

## Abstract

Cognitive impairment is a core feature of psychosis. Full cognitive assessments are not often conducted in routine clinical practice as administration is time-consuming. Here, we investigated whether brief tests of cognition could be used to predict broader neurocognitive performance in a manner practical for screening use in mental health services. We carried out a principal component analysis (PCA) to obtain an estimate of general cognitive function (*N* = 415). We investigated whether brief tests of memory accounted for a significant percentage of variation in the PCA scores. We used discriminant function analysis to determine if measures could predict classification as lower, intermediate or higher level of cognitive function and to what extent these groups overlapped with groups based on normative data. Memory tests correctly classified 65% of cases in the highest scoring group, 35% of cases in the intermediate group, and 77% of cases in the lowest scoring group. These PCA-derived groups and groups based on normative scores for the two tests were significantly associated (*χ*2 = 164.00, *p* < 0.001). These measures accurately identified three quarters of the low performing group, the group of greatest interest from the perspective of identifying those likely to need greater supports as part of clinical care. In so doing they suggest a potentially useful approach to screening for cognitive impairment in clinical services, upon which further assessment can be built if required.

## Introduction

Cognitive impairment in psychosis is associated with significant disability in social and occupational function^1–4^. These impairments are present well before the onset of psychosis, increase following a first episode of psychosis^3,5^, remain significant during the chronic stage of illness^6,7^, and are largely independent of variation in clinical symptom severity^4,8^. The severity of cognitive deficits (~1–3 standard deviations below non-clinical samples) vary by domain, the most prominent of which are related to memory, executive function, and social cognition^9–11^.

Despite the recognised importance of cognition in psychosis, full cognitive assessments are not often conducted in routine clinical practice as they are time-consuming, and availability of the expertise needed to carry out these assessments is limited. To overcome this, several brief screening measures have been developed. These include The Brief Assessment of Cognition in Schizophrenia^12^; the Screen for Cognitive Impairment in Psychiatry^13^; and the Brief Cognitive Assessment^14^. Despite the availability and brief administration of screening tools (taking from 15 to 30 min), they are not routinely used in clinical practice. While reasons for this poor implementation are multiple, one involves a sort of Catch-22. One the one hand, trained personnel required to carry out neurocognitive testing in psychosis are often unavailable, even in well-resourced clinical teams. On the other, when such personnel are available, they often favour a more detailed assessment to support clinical formulation, which these screening tools often don’t allow for.

One way to overcome this difficulty may be to identify cognitive subtests from a larger ‘gold standard’ battery. These subtests could then be used to both indicate whether further testing was clinically indicated, and, if so, could be included as part of that larger battery. In terms of inclusion criteria, such measures should not only show good psychometric properties and have good normative data, they should be focused on cognitive domains that show the largest deficits in psychosis, are sensitive to changes in treatment, and that are most strongly predictive of social and occupational outcomes. In terms of these criteria, tests of *episodic memory and working memory* demonstrate large cognitive impairments in psychosis, even in the early stages^5,15^, and both cognitive domains can be individually predictive of social and occupational function^3^. More recently, impairments in *social cognition* have emerged as a strong predictor of social and occupational function^4,11^, and are observed to mediate the relationship between global cognition and social and occupational function^16,17^.

In the present study we sought to determine whether brief tests of memory and social cognition could be used to predict overall neurocognitive performance in a manner practical for use in mental health services. A key requirement of these services is to understand and deliver interventions (including cognitively demanding psychosocial interventions, such as CBT) in a manner that is personalised to the needs of the individual so as to maximise treatment response. For example, identifying patients who experience significant cognitive difficulties may help to identify and respond to a need for cognitive remediation prior to starting other psychosocial interventions, and indeed for a psychosocial intervention to have a reduced emphasis on metacognitive skills.

To this end we investigated whether three commonly used measures of memory function and social cognition could (a) account for a significant percentage of variation on a general battery of tests (including general cognitive function, episodic and working memory, attention and executive function), and (b) could statistically predict classification as lower, intermediate or higher level of cognitive function based on the broader test battery. Finally, (c) we statistically compared individual classification as low/intermediate/high function based on the larger test battery with classification as low/intermediate/high based on available norms for the individual tests. In so doing, our hypotheses were that these brief measures could (1) be used to predict overall cognitive function based on PCA score, and (2) be used to reliably assign participants to clinically/cognitively meaningful groups in terms of intervention needs.

## Results

### Sample characteristics

The mean age of the sample was 43.02 (SD = 12.27, range 17–72), 65.56% of the sample were male. For diagnosis, 61% of the sample had schizophrenia, 12% had schizoaffective disorder, 16% had bipolar disorder, 6% had a psychotic disorder not otherwise specified, and 4% had depression with psychosis. For LM and LNS, data were available for 1131 cases. For RMET, data were available for 718 cases. 415 cases were included in the PCA across 5 measures.

### PCA-derived general cognitive function score

Prior to performing our PCA, the suitability of the data for the analysis was verified. The case-to-variable ratio was 83 (well exceeding the recommended minimum of 10)^18^, indicating adequacy of the sample size. The Kaiser-Meyer-Olkin sampling adequacy measure was 0.776 (the recommended value being ≥0.6), and the Bartlett’s Test of Sphericity was significant, both of which supported the factorability of the correlation matrix.

PCA was then used to estimate an overall cognitive factor score based on five different measures. Factor 1 explained 53% of the variance with factor loadings from 0.41 to 0.71. This factor was taken to provide an index of global cognitive performance, and *z*-scores derived for this factor were used to divide the sample based on the 33rd and 66th percentiles into 3 categories of performance (i.e. low, intermediate and high scores).

### Variance in global cognitive scores explained by logical memory and letter number sequencing

We carried out individual hierarchical multiple regression analyses to examine the variance in PCA derived global cognitive score explained by LM, LNS and RMET. In the first regression, after controlling for sex and age at time of assessment in step 1, Logical Memory accounted for 28% of the variance in PCA score(Δ *r*^*2*^ = 0.28, *p* < 0.001). In the second regression, LNS accounted for 29% of the variance in PCA score after controlling for sex and age in step 1(Δ *r*^*2*^ = 0.29, *p* < 0.001). In the third regression, RMET accounted for 12% of the variance in PCA score after controlling for sex and age (Δ *r*^*2*^ = 0.12, *p* < 0.001). As RMET had the lowest individual predictive value, we carried out a hierarchical multiple regression analysis to determine whether RMET added significant predictive value after LM and LNS were accounted for. After controlling for sex and age at time of assessment in step 1, LNS accounted for 27% of additional variance explained (Δ *r*^*2*^ = 0.27, *p* < 0.001) in step 2, in step 3 logical memory accounted for an additional 10% in variance explained (Δ *r*^*2*^ = 0.10, *p* < 0.001). In the final step, RMET did not significantly add predictive value (Δ *r*^*2*^ = 0.02, *p* = 0.05), and was therefore excluded from the measures used for further analysis.

### Discriminant function analysis to determine classification accuracy of cognitive ability

Discriminant Function Analysis (DFA) was carried out to determine if LM and LNS could predict classification into tercile groups of the PCA factor score. These assessments correctly classified 65% of cases in the highest scoring group, 35% of cases in the intermediate group, and 77% of cases in the lowest scoring group. A MANOVA revealed significant differences between factor score groups on LM and LNS scaled scores (*F*_(2, 408)_ = 70.24, *p* < 0.001) (See Table 1 for scaled scores for PCA groups). In summary, LNS and LM accurately identified three quarters of the low performing group, the group of greatest clinical interest from the perspective of identifying those likely to need greater supports as part of clinical care.

**Table 1:** Descriptive statistics for PCA factor score category.

Factor score category	LM scaled score mean (SD)	LNS scaled score mean (SD)
High	8.52 (2.96)	10.12 (2.88)
Intermediate	7.09 (3.04)	8.06 (2.85)
Low	4.37 (2.80)	5.50 (2.67)

### Comparison of PCA-derived global cognitive score groups to supportive triage groups

To determine the clinical utility of the LM and LN task to triage patients based on presumed cognitive function (based on general population scaled scores), we sought to categorise groups as low and likely impaired (scaled score ≤5; definite impairment, >2 SD below mean), intermediate (scaled score 6–8; possible impairment, between 1 and 2 SD below mean), and high (scaled score ≥9; likely unimpaired, <0.5 SD below mean or above). 34% of the sample fell within the unimpaired group, 34% of the sample in the intermediate group, and 32% of the sample in the impaired group. There were significant differences between these groups on the PCA cognitive factor score (*F*_(2, 409)_ = 145.68, *p* < 0.001). A Chi-Squared test revealed a significant association between triage group and PCA factor score group (*χ*^2^ = 164.00, *p* < 0.001). 65% of cases (*N* = 88 out of 136 total cases) for high PCA group (top tercile) overlapped with the high/unimpaired group (scaled score ≥ 9), 44% of cases (*N* = 58 out of 137 total) for intermediate PCA group overlapped with the intermediate triage group (scaled score 6–8), and 70% of cases (*N* = 94 out of 136 total) for low PCA group overlapped with the impaired triage group (scaled score ≥ 5) (See Table 2 for descriptive statistics).

**Table 2:** Descriptive statistics for supportive triage group and PCA factor score group.

Factor score category	LM&LNS averaged scaled score mean (SD)	Supportive triage group	LM&LNS averaged scaled score mean (SD)
High	9.32 (2.25)	High	10.59 (1.38)
Intermediate	7.58 (2.37)	Intermediate	7.30 (0.86)
Low	4.93 (2.08)	Low	3.88 (1.26)

### Differences in cognitive scores across different diagnoses

To investigate differences in LM and LNS scores associated with diagnosis and sex, an ANOVA was carried out which revealed a significant difference between groups for diagnosis (*F*_(4,980)_ = 12.01, *p* < 0.001), but not for sex (*F*_(1,980)_ = 0.23, *p* = 0.63). On both LM and LNS, those with bipolar disorder scored the highest, followed by depressive disorder, schizoaffective disorder, and those with schizophrenia scored the lowest. A linear regression was carried out to evaluate the association between age and LM and LNS scores, results revealed that although age was significantly associated the variance explained was small (*F*_(1, 1120)_ = 30.91, *p* < 0.001, *Adj r*^*2*^ = 0.03). In summary, patients could be categorised as unimpaired (scaled score ≥ 9), intermediate (possible impairment; scaled score 6–8) or impaired (scaled score ≤ 5) in a manner that made sense given their overall cognitive profiles as determined by our PCA score, not explained by sex, and that reflected diagnostic differences between groups (bipolar scoring highest, schizophrenia scoring lowest). See Tables 3 and 4 for descriptive statistics for LM and LNS for age, sex and diagnosis.

**Table 3:** Descriptive statistics for logical memory.

	*N*	Mean	SD
*Age range*			
16–24	93	7.51	3.19
25–29	107	6.84	3.51
30–34	125	6.49	3.35
35–44	297	7.07	3.28
45–54	308	6.81	3.72
55–64	216	6.50	3.84
65+	26	6.58	3.59
Overall	1172	6.84	3.54
Male	765	6.74	3.41
Female	399	7.04	3.78
*Diagnosis*			
Schizophrenia	627	6.30	3.57
Schizoaffective disorder	124	6.32	3.27
Bipolar disorder	171	8.09	3.30
Depressive disorder	39	7.38	3.57
Other	66	7.61	3.46
Non-affective psychosis	627	6.30	3.57
Affective psychosis	400	7.39	3.42

**Table 4:** Descriptive statistics for letter number sequencing.

	*N*	Mean	SD
*Age range*			
16–24	92	8.71	3.17
25–29	108	8.07	2.94
30–34	123	8.31	3.39
35–44	292	7.75	3.38
45–54	295	7.74	3.60
55–64	198	6.90	3.54
65+	23	6.17	3.68
Overall	1131	7.74	3.45
Male	738	7.79	3.49
Female	385	7.68	3.37
*Diagnosis*			
Schizophrenia	609	7.29	3.42
Schizoaffective disorder	121	7.77	3.58
Bipolar disorder	166	8.77	3.28
Depressive disorder	37	7.73	3.66
Other	64	8.64	3.70
Non-affective psychosis	609	7.29	3.42
Affective psychosis	388	8.34	3.50

### Difficulties with triaging based on cognitive function

A small percentage of participants (*N* = 123, 11% of total sample) scored high on one task and low on the other, i.e. categorised as group 1 (unimpaired) on one task and group 3 (impaired) on the other. To further investigate this group, *z*-scores (calculated from a larger sample of both patients and controls) from a number of cognitive tasks were plotted to determine the overall cognitive profile of these individuals. For those with impaired episodic memory (i.e. scaled score of 5 or less) and unimpaired working memory (i.e. scaled score of 9 or greater), an ANOVA revealed no significant group difference (*F*_(1,847)_ = 2.554, *p* = 0.110) in full-scale IQ (*M* = 96.52, SD = 15.78) when compared to either the ‘unimpaired’ group or the ‘possible impairment’ group (*M* = 92.67, SD = 18.99). However, for those with impaired working memory and unimpaired episodic memory, an ANOVA revealed a significant difference in full-scale IQ scores (*M* = 86.68, SD = 17.93) between this group versus the ‘unimpaired’ and ‘possible impairment’ groups (*F*_(1,8470)_ = 4.249, *p* = 0.04).

## Discussion

### Summary of findings

The purpose of the present study was to determine whether brief tests of memory and social cognition could successfully predict neurocognitive performance for people with psychosis in a manner practical for use in mental health services. Our analyses suggested that two brief memory tests can be used to reliably predict overall cognitive function (general cognitive function PCA score), and reliably classify an individual’s level of cognitive function (low, intermediate or high) so as to inform the provision of treatment supports. Our discriminant function analysis accurately classified a high percentage of cases in both the high (unimpaired) and low (impaired) groups, and to a lesser extent the intermediate group. When participants were classified based on normative data for these tasks, the resulting high (scaled score ≥9; unimpaired) and low (scaled score ≤5 impaired) groups significantly overlapped with the categories assigned based on the full battery. In noting the reduced accuracy of these tests in classifying intermediate performance, it is clear that a majority of those who fall into this category will require fuller assessment and that a brief assessment cannot be used to inform treatment selection. Our findings suggest that where someone has impaired LM function but unimpaired LNS function, this was less likely to impact general cognitive function and therefore less likely to be clinically important. By comparison, when someone has unimpaired LM function but impaired LNS function, this was likely to be associated with general cognitive impairment and therefore have wider implications for clinical care. Collectively, these findings suggests that these brief memory tests may represent a potentially useful and practical cognitive screening tool in clinical practice to triage service users for assessment or intervention.

The ability to correctly identify three out of every four cognitively impaired individuals and two out of every three unimpaired individuals using two brief triage tests has clinical utility for personalising treatment decisions about psychosocial interventions. In terms of treatment needs, it highlights those who are likely to require a cognitively supportive intervention (e.g. cognitive remediation) prior to commencing more cognitively challenging interventions (e.g. CBT). In addition, this form of cognitive screening has broader implications for treatment, as it allows multi-disciplinary teams to get a more holistic understanding of an individual’s presentation and difficulties, and may help clinicians to differentiate between cognitive impairment and negative symptoms that can have similar presentations (e.g. alogia, avolition)^19^. It allows teams to consider cognitive function in relation to the impact on overall function, given the relationship between cognitive function and social and occupational function^4^, and to consider other factors that might be contributing to impaired cognitive performance such as medication side effects^20^ or substance use. This can help to identify other treatment needs such as functional assessment, medication review, or occupational therapy. It may also help to explain the non-response of some individuals to psycho-social interventions such as CBT, which are often cognitively demanding.

### Limitations and future directions

While this study was based on a relatively large sample size, there was wide variation in illness duration. In addition, there was variability in study characteristics including treatment and diagnosis. The sample used in this study mainly consisted of patients with well established (chronic) illness; thus, it would be useful to estimate the predictive value of memory for overall cognitive function in first-episode psychosis or early psychosis samples specifically, to determine if a different pattern of cognitive impairment is evident in early psychosis versus more chronic stages of illness. Furthermore, this study focused on estimating the predictive value of memory and social cognition; it may be informative to alternatively consider the value of other cognitive domains also, which was beyond the scope of our study. Notwithstanding these limitations, our findings highlight potential avenues for future research. It would be useful to consider the relative predictive value of memory for overall cognitive function compared to other cognitive domains such as processing speed or executive function.

Subtests from the Wechsler Memory Scale (WMS) are protected in many jurisdictions and require a high level of expertise to interpret (i.e. clinical psychologist or closely related field with training in the ethical administration, scoring, and interpretation of clinical assessments related to the intended use of the assessment). However, these restrictions are intended for use of the full battery and interpretation for the assessment of memory function. We propose the use of two subtests from the WMS only as a brief screening tool to indicate general cognitive function and to triage service users. In community mental health teams clinical psychologists, psychologists in clinical training, or other healthcare professionals with expertise and/or training in administering and scoring cognitive assessments or supervised clinical experience can administer the subtests. However, the input of a clinical psychologist may be needed to make decisions about further assessment and/or treatment arising from this screening. Although this may not satisfy professional standards for test administration for the original intended use of a comprehensive memory assessment, from a proof of concept point of view, we suggest our findings provide evidence of the usefulness of brief memory tests only for the purpose of screening. The credential requirements for test administrators would be more relevant at the stage of more comprehensive, formal assessment following the proposed informal screening stage.

Finally, it was noteworthy that once memory function was accounted for, social cognitive function did not appear to contribute further to prediction of general cognitive performance. Social cognition has been identified as particularly important for predicting social and occupational function in both early stage^4^ and chronic illness^2^. In the present study, the focus was on predicting cognitive performance rather than social and occupational function and it would appear that once memory domains closely linked to global cognitive performance were accounted for, social cognition did not significantly contribute to the predictive model for general cognitive performance, at least as measured by the total outcome score (of 36) from the Eyes of the Mind task, a widely used measure of mental state decoding. It is possible that studies including other measures of theory of mind, subfields of mental state decoding, or other domains of social cognition (e.g. emotion recognition) may have greater predictive power for general cognitive function.

## Conclusions

The findings from this study suggests that it is possible to identify cognitive impairment using brief screening measures taken from larger batteries, so as to predict overall neurocognitive performance in typical clinical settings. These brief measures are easy to administer and score, and thus are more likely to be feasible for clinicians to administer and well tolerated by service users. Furthermore, these neuropsychological tests are already used widely in research and clinical settings, and are well normed.

From a health economics perspective, used as cognitive screening tool these measures have the potential to be effective and efficient in identifying cognitive treatment needs within a service and targeting interventions accordingly. In particular, they may be useful for identifying clients whose cognitive difficulties present a barrier to engaging/benefitting from other psychosocial interventions (e.g. CBT) and who may benefit from prioritising these difficulties (e.g. using cognitive remediation therapy). This may also be useful in an early intervention context, given the difficulties in differentiating between cognitive versus other negative symptoms of psychosis (e.g. alogia and avolition), and considering cognitive impairment can be present at initial stages of illness.

In conclusion, identification of cognitive impairment in the early phases of treatment is important but is often challenging for clinicians and not adequately assessed. The brief measures proposed here may represent a suitable and cost-effective tool for the screening of cognitive impairment in clinical settings. These measures take <10 min to complete, and are therefore easily incorporated into initial assessments so that cognitive difficulties can be considered in the initial phases of treatment. So doing would allow more efficient allocation of resources in services, could inform clinician understanding of the overall presentation of illness, and ultimately improve individualised treatment.

## Methods

### Participants

Participants (*N* = 415) were selected using an existing neurocognitive dataset of patients with psychosis. Participants were recruited from outpatient community mental health teams, with a majority recruited from Dublin and Galway. Relevant university and hospital research ethics committees (Dublin and Galway) provided ethical approval for the studies included. Diagnosis was confirmed by a trained psychiatric research nurse based on a Structured Clinical Interview for DSM-IV (SCID) and review of all available clinical information. Inclusion criteria were that participants were aged between 18 and 65 years, had a history of psychosis, were community-based and clinically stable (in the opinion of the treating team), fluent in English and able to provide written informed consent. Exclusion criteria included history of organic impairment, head injury with loss of consciousness >5 min duration, neurological disorders, intellectual disability, or drug abuse in the preceding month.

### Selected measures of cognition

#### Measures included in principal component analysis

Five measures were included in our Principal Component Analysis (PCA) (PCA methods described in more detail below). Attention was assessed using the Sustained Attention to Response Task (SART; Robertson et al., 1997^21^). The SART is a Go/No-Go sustained task in which participants viewed the numbers 1–9 presented in a fixed order, and are required to button press for each number except the number 3. Participants also completed two subtests from the Wechsler Adult Intelligence Scale–Third Edition (WAIS-III; Weschler, 1997^22^): Similarities (verbal comprehension) and Matrix reasoning (perceptual organization) as indices of general cognitive function. In addition, participants completed the paired associates learning (PAL) subtest from the Cambridge Neuropsychological Test Automated Battery (CANTAB, 2019^23^), which assesses visual associative memory, and the spatial working memory (SWM) subtest from the CANTAB, which assesses visual working memory.

### Brief measures of memory and social cognition used to predict overall neurocognitive performance

Memory function was assessed using the Logical Memory (LM; verbal episodic memory) and Letter Number Sequencing (LNS; verbal working memory) tasks from the WMS – 3rd Edition (WMS-III; Wechsler, 1997^24^). LM (immediate recall) involves oral presentation of two standardised short stories, immediately after which participants are asked to recall the respective story verbatim. In the LNS task, participants are presented with a string of numbers and letters of increasing length and are asked to recall the numbers in ascending order, followed by the letters in alphabetical order.

Social cognition was assessed with the Reading the Mind in the Eyes Task (RMET; Baron-Cohen et al., 2001^25^). This measures the ability to infer mental states of others from restricted pictures of their eyes. Participants are shown 36 black and white static photos and are asked to choose one of four words that best describes the mental state expressed in the picture.

### Statistical analysis

To determine if three brief measures could be used to predict overall cognitive function and reliably assign participants to clinically meaningful groups based on cognitive function, we carried out statistical analyses in 4 steps. Firstly, we carried out PCA to reduce data to obtain a single primary factor score for overall general cognitive function. Using these scores, the sample was then divided into terciles of low, intermediate and high cognitive function. Secondly, we carried out linear regression analyses to determine the predictive value of each measure individually to explain variance in PCA factor score. Thirdly, we used discriminant function analysis to determine if our brief measures could be used to correctly classify individuals into the low, intermediate and high performance groups based on the PCA-derived score. In the last step, we re-grouped the samples based on the available standardised scores for the brief measures used and statistically compared this classification with that obtained from the PCA-derived tercile scores using a chi-square test.

### Principal component analysis

The PCA was based on the following subtests: Paired Associate Learning (PAL; CANTAB) total errors score (visual memory), Spatial Working Memory (SWM; CANTAB) between errors scores (working memory), SART total omission errors, Similarities and Matrix Reasoning subtests (WAIS-III). These measures were included as the most data were available for these measure and the potential for scores to contribute to the explained variance of an overall cognitive factor score. The scores from these five cognitive assessments were subjected to a PCA for the purposes of data reduction so as to obtain one primary (global) factor score. This was achieved using the first principal component score from an unrotated factor solution to explain maximum amount of variance with the minimum number of factors. Individual *z*-scores for this principal component were then used to categorise the sample based on the 33rd and 66th percentiles as “low” (<33rd), “intermediate” (34th–66th) and “high” (>66th) performance scores. The percentile scores are ranked based on data from the psychosis sample so may not necessarily reflect general population scores.

### Regression analysis

Prior to carrying out discriminant function analysis, linear regressions were carried out to determine the predictive value of each measure individually to explain variance in PCA factor score, to ensure each measure was adding predictive value and the inclusion of all three measures was necessary for further analysis to contribute to accuracy of the measures in classification of groups. We carried out three separate hierarchical multiple regressions with logical memory, letter number sequencing, and RMET as the predictor variables and PCA factor score as the dependent variable, sex and age at time of assessment were controlled for in step 1, and each measure was entered in step 2. Finally, we carried out an overall hierarchical multiple regression with all three measures entered in subsequent steps after controlling for sex and age in the first step.

### Discriminant function analysis

Whereas regression analyses estimate the variance explained by measures for a continuous variable (PCA score), discriminant function analysis estimates which categorical group an individual belongs to, as well as providing information about the accuracy of this classification. Discriminant function analysis was carried out to determine if LM and LNS scores could be used to accurately classify participants into high, intermediate and low PCA groups. We then compared the factor score groups on LM and LNS scaled scores using a MANOVA.

### Comparison of groups based on PCA-derived global scores versus standardised scores for individual tests

Finally, we grouped participants based on standardised scores for the general population. This step was to determine the clinical utility of the LM and LNS tasks to triage patients based on presumed cognitive function. Specifically, we sought to categorise groups using a supportive triage system of three groups: low (definite impairment), intermediate (possible impairment), or high (unimpaired cognitive function) based on the scaled scores for these tasks. To do this, the groups were defined using scaled scores as follows: group 1 (≥9; unimpaired), group 2 (6–8; possible impairment), group 3 (≤5; definite impairment). To further investigate those who were categorised as high (group 1) on one task and low (group 3) on the other task and vice versa, *z*-scores (calculated from a larger sample of both patients and controls) from a number of cognitive tasks were plotted to determine the overall cognitive profile of these individuals. We also conducted individual ANOVAs to compare general cognitive function (IQ scores) between high/low groups and ‘unimpaired’ or the ‘possible impairment’ groups. We then compared the triage (scaled score) groups to our PCA general cognitive score groups (low, intermediate, high) using chi-square test to determine if these categories corresponded. For this analysis, we averaged LM and LNS scores to create one variable to use for categorising. Finally, we compared groups for diagnosis and sex differences.

## Data Availability

The datasets generated during and/or analysed during the current study are not publicly available as the data was collected over a prolonged period of time across multiple studies and public availability of the data was not included in ethical approval, but are available from the corresponding author on reasonable request.
